# COPA and SLC4A4 are Required for Cellular Entry of Arginine-Rich Peptides

**DOI:** 10.1371/journal.pone.0086639

**Published:** 2014-01-28

**Authors:** Tomoyuki Tsumuraya, Masayuki Matsushita

**Affiliations:** Department of Molecular and Cellular Physiology, Graduate School of Medicine, University of the Ryukyus, Okinawa, Japan; Stanford, United States of America

## Abstract

Cell-penetrating peptides (CPPs) have gained attention as promising tools to enable the delivery of various molecules in a non-invasive manner. Among the CPPs, TAT and poly-arginine have been extensively utilized in numerous studies for the delivery of functional proteins, peptides, and macromolecules to analyze cellular signaling. However, the molecular mechanisms of cellular entry remain largely unknown. Here, we applied siRNA library screening to identify the regulatory genes for the cellular entry of poly-arginine peptide based on microscopic observation of the entry of fluorescent peptides in siRNA-treated cells. In this screening, we identified the cell membrane gene SLC4A4 and the trafficking regulator gene COPA, which also plays an important role in early endosome maturation. These results demonstrated that cellular entry of poly-arginine requires at least two different steps, probably binding on the cell surface and endosomal entry. The identification of genes for cellular entry of poly-arginine provides insights into its mechanisms and should further aid in the development of highly efficient cell-penetrating peptides.

## Introduction

The cell membrane is the barrier that separates the inside and outside of cells, and highly hydrophilic substances such as proteins, peptides, and nucleic acids are not usually transmitted through the cell membrane. Recently, using cell-penetrating peptides (CPP) as vectors, methods have been developed for introducing membrane-impermeable molecules into cells. Among these CPPs, the cationic TAT peptide derived from human immunodeficiency virus type 1 (HIV-1) [1], [2], [3] and the arginine-rich peptide (9R–11R) are the most frequently used [4], [5], [6]. These CPPs have great potential for the delivery of macromolecules such as proteins, peptides, RNA, and imaging compounds in applications such as cell culture, animal disease models [7], [8], [9]. Therefore, numerous studies have been carried out to investigate the cellular entry mechanisms of cationic CPPs. Early studies suggested that the membrane penetration of CPPs is a non-energetic, temperature-independent, and non-endocytic process, and thus CPPs cross the lipid bilayer directly [10]. However, the fixation of cells during these studies drastically enhanced the permeability of cell membranes, resulting in artificial observations [11]. Subsequently, increased numbers of studies have shown that cellular uptake of cationic CPPs requires proper temperature and energy expenditure, and therefore energy-dependent processes are the major route for membrane translocation [12], [13], [14]. In addition, negatively charged heparan sulfate proteoglycans (HS) on the cell surface play an important role in binding to cationic CPPs, and cellular uptake occurs by the processes of endocytosis or macropinocytosis [15], [16], [17]. Functional delivery of macromolecules fused with CPPs requires endocytotic vesicle escape for cytosolic localization and thus proper functioning in cells. To enhance peptide escape from vesicles, several studies have used pH sensitive HA peptide, cathepsin peptide, or photosensitive methods to destroy the vesicles. These studies suggest that the cellular entry of CPPs has 3 steps: initial binding to a cell surface receptor, entry by endocytotic processes, and vesicle escape [16], [18], [19], [20]. However, a recent study showed that heparan sulfate proteoglycan is not required for functional delivery of TAT peptide, which may have another cellular entry route for functional delivery [21], [22], [23]. Despite numerous studies, the molecular mechanisms of CPP cellular entry remain largely unknown. To elucidate these molecular mechanisms, the identification of genes involved in the cellular uptake of cationic peptides is required in order to enhance their efficiency. Here, we show that a loss-of-function siRNA library screen allowed for systematic identification of genes required for cellular entry of FITC-9R. In the present study, we identified potential regulator genes involved in the cellular entry of 9R peptide using a genome-wide siRNA library containing 991 human membrane-associated genes.

## Results

### siRNA Library Screening

**Figure 1:**
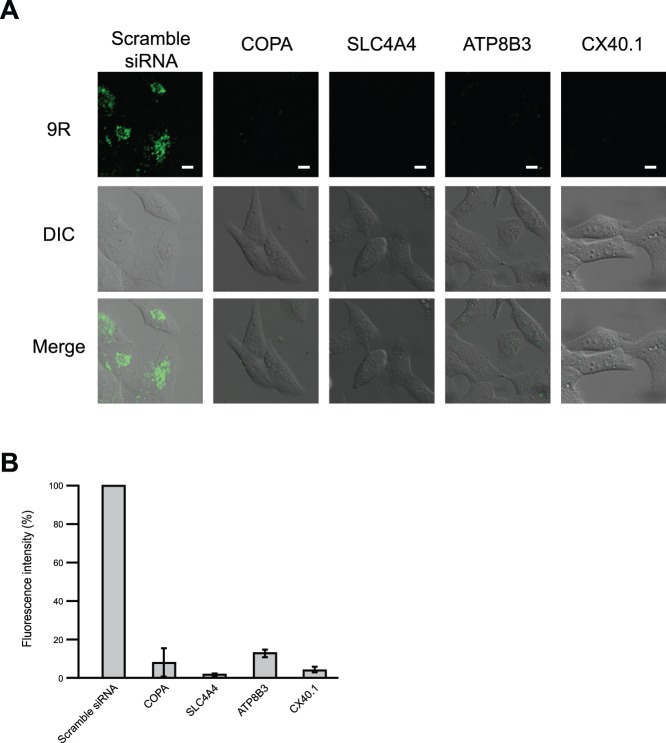
Examination of FITC-9R entry in siRNA treated cells. Analysis of FITC-9R uptake in HeLa cells transfected with siRNAs against COPA, SLC4A4, ATP8B3, and CX40.1. (A) FITC-9R uptake of HeLa cells transfected with 4 individual siRNAs that were selected by primary screening. Scale bars = 10 µm. (B) Fluorescence intensity was measured by MetaMorph. Error bars represent SD from three independent experiments.

To identify genes that are involved in FITC-9R internalization into HeLa cells, we performed genome-wide screening using an siRNA library. The siRNA library used in this study was divided into two sub-libraries, a membrane transporter and a transporter library, which contained 695 and 296 siRNAs, respectively. HeLa cells were cultured in 96-well format glass bottom plates and transfected with each siRNA. After 72 h, we exposed the cells to 10 µM FITC-9R for 1 h, and changed the medium followed by observation under a confocal microscope. In first screen, we identified 21 genes that decreased the uptake of FITC-9R compared with mock-transfected cells by laser confocal microscope observation. We did the same operation again for these 21 siRNAs, and found that 4 genes decreased the uptake of FITC-9R. These 4 genes were COPA, SLC4A4, ATP8B3, and CX40.1 (Fig. 1A). The fluorescence intensity of HeLa cells was measured by MetaMorph (Olympus, Tokyo, Japan), which showed that COPA, SLC4A4, ATP8B3, and CX40.1 siRNAs reduced the uptake of FITC-9R compared with scrambled siRNA (Fig. 1B).

### Validation of Identified Genes by siRNA Library Screening

**Figure 2:**
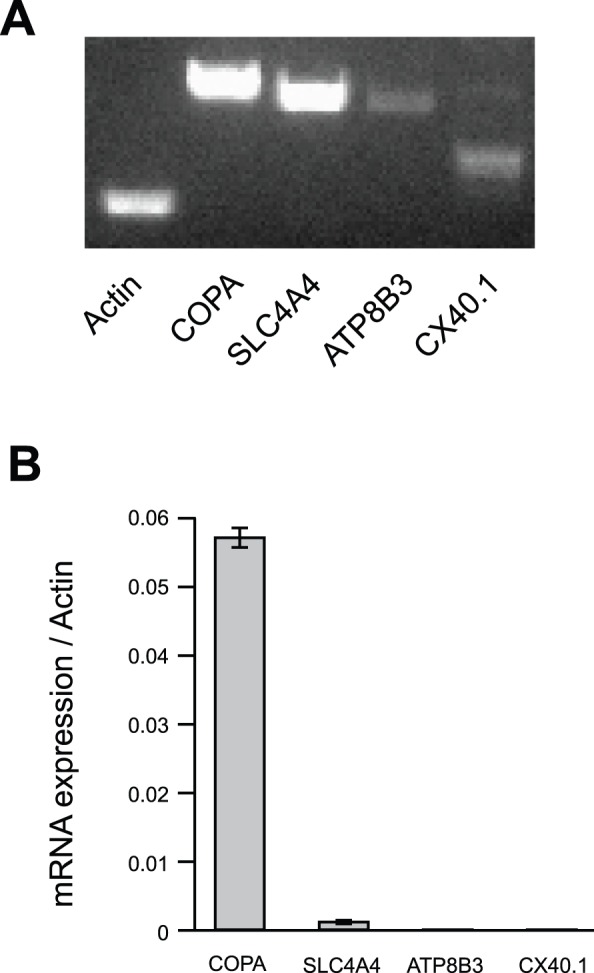
Expression of 4 identified genes in HeLa cells. (A) Agarose gel electrophoresis showing PCR amplification products for COPA, SLC4A4, ATP8B3, and cX40.1. All PCR products were detected at the predicted sizes. (B) Expression levels of COPA, SLC4A4, ATP8B3, and cX40.1 mRNA were determined by real-time RT-PCR. Error bars represent SD from four independent experiments.

**Figure 3:**
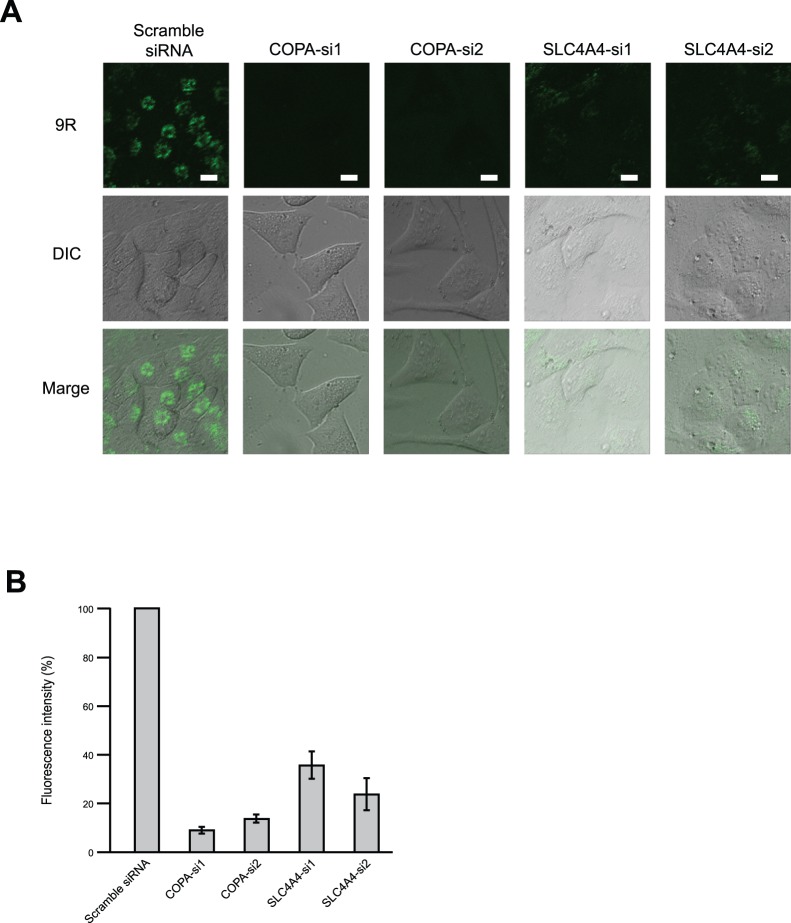
Confirmation of gene specificity of siRNAs. (A) RNA interference for COPA and SLC4A4. Interference of each gene was executed by two distinct gene-specific siRNAs. HeLa cells were cultured with each siRNA for 72 h. FITC-9R was added 1 h at 37°C before observation, and cells were observed with confocal microscopy. Scale bars = 10 µm. (B) Fluorescence intensity was measured by MetaMorph. Error bars represent SD from three independent experiments.

COPA and SLC4A4 were confirmed to be expressed in HeLa cells, but ATP8B3 and CX40.1 expression was very low (Fig. 2A, B). Thus, we focused on COPA and SLC4A4 for further investigation. In order to exclude the possibility of off-target effects, we prepared additional siRNA target sequences against COPA and SLC4A4. Knockdown of each gene was executed by each of two distinct gene-specific siRNAs, and each was confirmed to reduce the uptake of FITC-9R (Fig. 3A). The fluorescence intensity of HeLa cells was measured by MetaMorph, which showed that COPA siRNA1, COPA siRNA2, SLC4A4 siRNA1, and SLC4A4 siRNA2 reduced the internalization of FITC-9R compared with scrambled siRNA (Fig. 3B). In addition, we examined the inhibitory effect of cellular entry of FITC-9R with COPA and SLC4A4 mRNA knockdown in other type of cell lines (Fig. S1). These cell lines treated with COPA and SLC4A4 siRNA suppressed the uptake of FITC-9R. We confirmed the knockdown efficiency of COPA and SLC4A4 siRNA by real-time PCR (Fig. S2).

**Figure 4:**
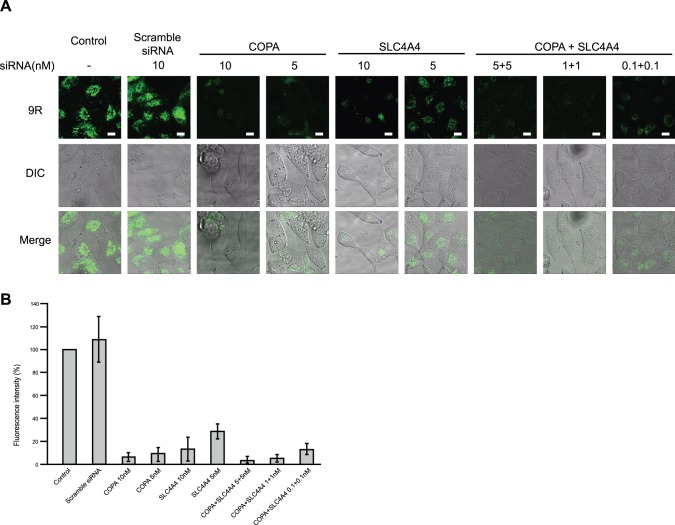
Double knockdown of COPA and SLC4A4 in HeLa cells. (A) HeLa cells were transfected with COPA and/or SLC4A4 siRNAs and cultured for 72 h. Then FITC-9R was added to cells and incubated for 1 h at 37°C. Cells were observed with confocal microscopy. Scale bars = 10 µm. (B) Fluorescence intensity was measured by MetaMorph. Error bars represent SD from three independent experiments.

We also found that double knockdown with co-transfected COPA and SLC4A4 siRNAs suppressed the uptake of FITC-9R at lower concentrations than with the single-transfected siRNAs (Fig. 4A, B).

### Localization of COPA and SLC4A4 with FITC-9R

**Figure 5:**
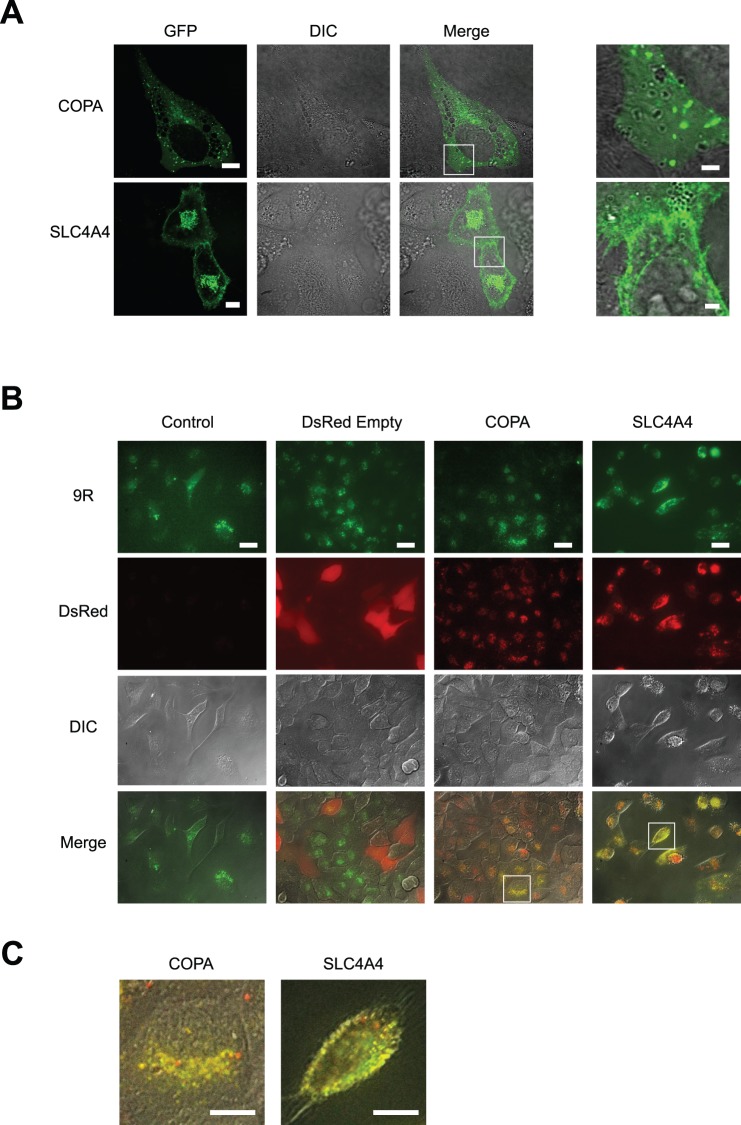
COPA and SLC4A4 co-localized with FITC-9R. (A) Confocal images of EGFP-COPA and EGFP-SLC4A4 localization in HeLa cells. Scale bars, 10 µm. Right Panel, high magnification merge images. Scale bars = 2 µm. (B) Confocal microscopy images of double fluorescence imaging show the co-localization of FITC-9R with COPA or SLC4A4 in HeLa cells. HeLa cells identified by DIC (differential interference contrast), and signaling with FITC-9R (9R) (green) were also positive for COPA (red), and co-localization was evident when images were merged (yellow). Similarly, the expression of SLC4A4 co-localized with FITC-9R is shown. pDsRed empty vector was used as a negative control and did not show co-localization with FITC-9R. Scale bars, 20 µm. (C) High magnification images from Fig. 5A. Scale bars = 10 µm.

First, we examined the cellular localization of SLC4A4 and COPA genes. GFP-fused COPA and SLC4A4 were expressed in HeLa cells and observed by fluorescence microscopy. GFP-COPA was observed as a granular-like signal (Fig. 5A), as shown in previous studies [24]. COPA is one of the seven non-clathrin-coated vesicular coat subunits that form the “coatomer”, which plays a role in membrane transport between the endoplasmic reticulum and the Golgi apparatus [25], [26]. Non-clathrin-coated vesicular coat subunits also include F-COPI and B-COPI (the COPA gene encodes for the α subunit of the B-COPI complex). We also investigated the localization of SLC4A4. SLC4A4 is an electrogenic sodium/bicarbonate co-transporter at the cell membrane and is thought to regulate intracellular pH [27], [28], [29]. As shown in Fig. 5A, GFP-SLC4A4 localized at the cell membrane. Since 9R is conjugated with FITC, we created DsRed-COPA and DsRed-SLC4A4 expression vectors for double fluorescent imaging. Then, we examined the co-localization of FITC-9R with DsRed-COPA and DsRed-SLC4A4 in HeLa cells. COPA and SLC4A4 signals overlapped with FITC-9R (Fig. 5B, C).

### COPA and SLC4A4 Regulate TAT Peptide Uptake

**Figure 6:**
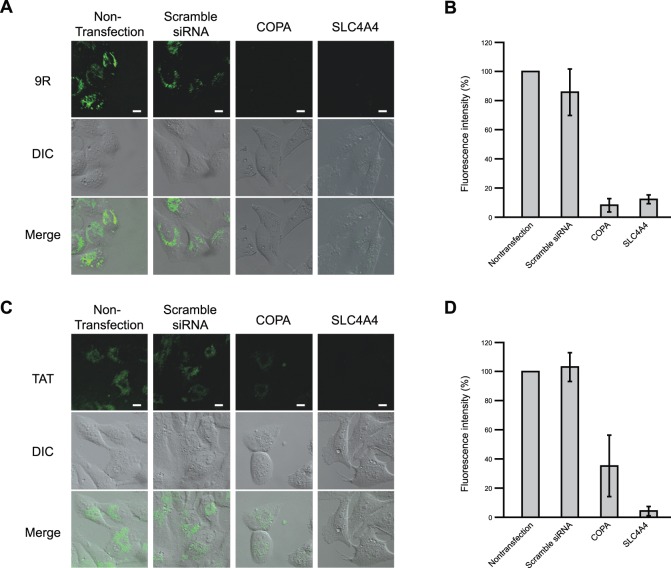
Cell uptake effect of FITC-9R and FITC-TAT in COPA or SLC4A4 siRNA-treated cells. HeLa cells were transfected COPA or SLC4A4 siRNAs, and after 72-9R or FITC-TAT were added and incubated for 1 h at 37°C. Cells were observed with confocal microscopy (A, C). Scale bars = 10 µm. Fluorescence intensity of FITC-9R and FITC-TAT were measured by MetaMorph (B, D). Error bars represent SD from three independent experiments.

The Tat protein transduction domain (TAT), a small region of HIV-1 Tat (trans-acting activator of transcription) corresponding to residues 47-YGRKKRRQRRR-57, is responsible for translocating the protein across the plasma membrane [1]. A number of studies have reported the strong cellular entry property of this short peptide after fusion with various proteins both *in vivo* and *in vitro* [2], [3]. To investigate the entry mechanism of TAT with regards to COPA and SLC4A4, we investigated FITC-TAT cellular entry with COPA or SLC4A4 siRNA-treated HeLa cells. HeLa cells were incubated with COPA or SLC4A4 siRNA for 72 h, and then treated with 10 µM 9R-FITC for 1 h (Fig. 6A, B). We found that COPA and SLC4A4 knockdown also inhibited the cellular entry activity of TAT (Fig. 6C, D).

## Discussion

Although the exact cell entry mechanisms of CPPs remain unclear, macropinocytosis or endosomal pathways are thought to be the main routes of internalization of CPPs. For example, the TAT peptide belongs to the cationic CPPs and is one of the most widely used for delivery of macromolecules into various cells. The cell-penetrating activity of TAT requires its interaction with cell surface heparan sulfate through the binding of the negatively charged sulfate group [13], [14], [15]. The basic amino acid within TAT recognizes the negatively charged heparan sulfate proteoglycan, and then this interaction triggers TAT cell entry through macropinocytosis. Argine-rich peptide (9R-11R) also shares the same cell entry mechanism with TAT [17]. However, recent studies have shown that cell delivery of functional molecules with TAT occurs in the absence of heparan sulfate and sialic acid [21].

Intensive studies have been performed to clarify the cell entry mechanisms of CPPs; however, the molecular mechanisms are still controversial. To investigate the initial step of cationic CPP transduction or penetration on the cell membrane, we applied an siRNA library screen to identify genes that quantifiably affected the uptake of FITC-9R by fluorescence microscopy. With this screen, we identified two membrane-associated genes, COPA and SLC4A4. Knockdown of these genes significantly reduced the cellular entry of 9R and TAT peptide in HeLa cells (Fig. 6). The SLC4A4 gene encodes the electrogenic bicarbonate cotransporter (NBCe1). The Na^+^-HCO_3_^−^ cotransporters (NBC) encoded by the SLC4 gene family are recognized as important mechanisms for HCO_3_^−^ flux in many types of cells. NBCe1 is glycosylated at the third extracellular loop, and that glycosylation may be required for the initial cationic CPP binding step at the cell membrane [30]. NBCe1 also localizes at the plasma membrane and undergoes constitutive endocytosis according to previous studies [31], [32]. These functional studies and our present results suggest that large amounts of cationic peptides bind SLC4A4 (NBCe1) at the cell membrane and enter by endocytosis in HeLa cells, HepG2, U-87 MG and HEK293 (Fig. S1). Other types of cells may have distinct cell membrane proteins for the entry of cationic peptides.

We also identified the COPA gene (encoding the α subunit of the B-COPI complex), which plays a crucial role in transport from the cis-Golgi to the ER and intra-Golgi trafficking [25], [26]. A recent study also showed that the COP complex plays non-canonical roles such as endosome maturation, apoptosis, and cell division through the regulation of membrane trafficking functions [33], [34]. Our results showed that TAT and 9R share these two molecules for cellular entry (Fig. 6). Cationic amino acid composition is crucial for these CPPs.

These two identified genes are involved in endocytosis and early endosome maturation. We concluded that, for the most part, the cellular entry of 9R requires an endocytosis-like mechanism. However, our studies did not address the mechanism of endosomal escape or functional protein delivery because of the limitations of the screening method. A combination of siRNA library screening and TAT-CRE transduction in a floxed system should solve the molecular mechanism of functional delivery of these peptides. Although the escape mechanism from endosomal vesicles is still missing, these identified genes should shed light on the cellular entry mechanisms of cationic CPPs, and will help lead to the creation of more efficient delivery peptides.

## Materials and Methods

### Peptide Synthesis

FITC-conjugated peptides were synthesized by Sigma-Aldrich. Peptides were purified by preparative reversed-phase HPLC and were >95.9% pure, with the expected amino acid composition and mass spectra.

### Cell Culture

Cells were grown in DMEM (Invitrogen), with 10% standard FBS (Invitrogen),100 U/ml penicillin, and 100 µg/ml streptomycin (Invitrogen) added, at 37°C in a humidified atmosphere of 5% CO_2_. Experiments were performed by first exchanging the medium with antibiotic-free medium before cell treatments.

### siRNA Library

We used a human genome-wide siRNA library (siPerfect, containing small double-stranded RNAs against 9,869 human genes obtained from Sigma-Aldrich, Tokyo, Japan). This library consists of nine sublibraries: kinase, phosphatase, ion channel, membrane transporter, transporter, transcription factor, receptor, nucleic acid binding, and ion binding. The transporter and membrane transporter sublibraries were used in the primary screening. The siRNA target sequences for COPA, SLC4A4, ATP8B3, and CX40.1 are COPA : CCATTGATCCCACTGAGTTCA, SLC4A4 : GCCACACATCATGCTGATAAA, ATP8B3 : GGGAGGAACGGGTCTACCAGG, CX40.1 : CGGGGCGACCCGTCTACCAGG. We also obtained additional gene-specific siRNAs (FlexiTube siRNA, QIAGEN) for secondary screening. The siRNA target sequences for COPA siRNA1-2 and SLC4A4 siRNA 1-2 are COPA siRNA1: CTGGCGCATGAATGAATCAAA, COPA siRNA2 : CACACGGGTGAAGGGCAACAA, SLC4A4 siRNA1 : CCGGCTTTGTTGGTCACTATA, SLC4A4 siRNA2 : CCGGCTTTGTTGGTCACTATA.

### RNA Interference

For analysis of putative proteins that mediate the transduction of 9R, cells were seeded into 96-well plates (5×10^3^ cells/well) and transfected in serum-containing medium (without antibiotics) with 50 nM siRNAs using DharmaFECT Transfection Reagents (Thermo Scientific). After 72 h of culture, cells were incubated with 10 µM FITC-9R for 1 h at 37°C. Cell fluorescence was observed by confocal microscopy (Leica DMI6000 CS).

### Quantitative Fluorescent Image Analysis

Fluorescence intensities of the microscopic images obtained from the HeLa cells incorporating 10 µM FITC-9R were measured with MetaMorph software Version 6 (Olympus, Japan). Living cells exhibiting normal nuclear morphology were considered with respect to acquisition of fluorescence intensity per cell; furthermore, analysis was performed utilizing the entire soma of individual cells as the region of interest (ROI). The fluorescence intensity of 3 cells was counted, and three sets of experiments were conducted. Background fluorescence intensity was subtracted from all experiments.

### Real-time Quantitative Reverse Transcriptase-PCR

Total RNA was extracted with TRIzol (Invitrogen) following the manufacturer’s instructions. A SmartSpec Plus Spectrophotometer (Bio-Rad) was used to assess the quality of the RNA. Five hundred ng of total RNA was then reverse transcribed into cDNA with random primers with the PrimeScript RT reagent kit (TaKaRa). PCR primers used were 5′-CCACTATCAGAATGCCCTATACC-3′ (forward) and 5′-CCACAAACCCATCTTCATCC-3′ (reverse) for COPA, 5′-ACTGGCCCCACAGTATTTGC-3′ (forward) and 5′-ACCTCGGTTTGGACTTGTTG-3′ (reverse) for SLC4A4, 5′-CGCAGAGTCCTTCTTCGTCTTC-3′ (forward) and 5′-ACTTGTTCCAGAGGTAGGGGTTC-3′ (reverse) for ATP8B3, 5′-AGTCCCTGCTGATGCTGTTC-3′ (forward), ′-CACCTCACTCTCATCCTCATCC-3′ (reverse) for CX40.1, and 5′-CCTCATGAAGATCCTCACCGA-3′ (forward) and 5′-TTGCCAATGGTGATGACCTGG-3′ (reverse) for β-actin.

Real-time quantitative reverse transcriptase-PCR was performed with a LightCycler1.5 (Roche) with SYBR Premix Ex Taq (TaKaRa) following the manufacturer’s instructions. β-actin was selected as an internal control for RNA input and reverse transcription efficiency. All PCR reactions were done in duplicate for both target genes and the internal control. After controlling for equal PCR efficiency of target genes and internal controls, relative gene expressions were calculated.

### Generation of Fluorescent COPA and SLC4A4 Plasmids

pDsRed-Monomer-C1 (TaKaRa) was used for generating plasmid vectors according to the manufacturer’s instructions. In brief, COPA cDNA and SLC4A4 cDNA were amplified by PCR. Primers containing XhoI (5′) and EcoRI (3′) linkers were synthesized as follows. COPA: 5′-CTCGAGCTATGTTAACCAAATTCGAG-ACCAA-3′ (forward), 5′-GAATTCTTAGCGAAACTGCAGAGGACTGA-3′ (reverse); SLC4A4∶5′-CTCGAGCTATGTCCACTGAAAATGTGGAAGGGAA-3′ (forward), 5′-GAATTCTCAGCATGATGTGTGGCGTTCAAGGAA-3′ (reverse).

The identities of the genes were confirmed by sequencing, and they were subsequently cloned into the same restriction sites in vector pDsRed-Monomer-C1 that contains a monomeric mutant derived from the tetrameric *Discosoma* sp. red fluorescent protein.

### Analysis of Protein Localization

Uptake and intracellular localization of FITC-9R and pDsRed peptides were visualized in live cells by confocal microscopy. HeLa cells were transfected with recombinant vectors COPA-pDsRed and SLC4A4-pDsRed with Lipofectamine LTX Reagent (Invitrogen) for 48 h, and subsequently treated with 10 µM FITC-9R for 1 h. The cells were washed out gently, and we obtained fluorescent images.

## Supporting Information

Figure S1 **Examination of FITC-9R entry in COPA or SLC4A4 siRNA treated cells.** Cells were transfected COPA or SLC4A4 siRNAs (Sigma Aldrich), and after 72 h, FITC-9R was added and incubated for 1 h at 37°C. HeLa (A), HepG2 (B), U-87 MG (C), and HEK293 (D) cells were observed with confocal microscopy. Scale bars = 10 µm. Fluorescence intensity of HeLa (E), HepG2 (F), U-87 MG (G), and HEK293 (H) was measured by MetaMorph. Error bars represent SD from three independent experiments.
(DOC)

Figure S2 **siRNA knockdown efficiency in 4 cell lines.** Analysis efficiency of siRNA against COPA and SLC4A4 in 4 cell lines. COPA siRNA (A–D) and SLC4A4 siRNA (E–F) were transfected in HeLa (A, E), HepG2 (B, F), U-87 MG (C, G), and HEK293 (D, H). After 24 h, mRNA expressions were determined by real-time RT-PCR. Error bars represent SD from three independent experiments.
(DOC)
