# Supplementary material for: COPA and SLC4A4 are Required for Cellular Entry of Arginine-Rich Peptides
Source: PLoS One. 2014 Jan 28;9(1):e86639. doi: 10.1371/journal.pone.0086639 (PMC3904941; doi:10.1371/journal.pone.0086639)
Supplement: Figure S2 — siRNA knockdown efficiency in 4 cell lines. Analysis efficiency of siRNA against COPA and SLC4A4 in 4 cell lines. COPA siRNA (A–D) and SLC4A4 siRNA (E–F) were transfected in HeLa (A, E), HepG2 (B, F), U-87 MG (C, G), and HEK293 (D, H). After 24 h, mRNA expressions were determined by real-time RT-PCR. Error bars represent SD from three independent experiments. (DOC) [file pone.0086639.s002.doc]

**FIgure S2**
